# Use of Health Belief Model–Based Deep Learning Classifiers for COVID-19 Social Media Content to Examine Public Perceptions of Physical Distancing: Model Development and Case Study

**DOI:** 10.2196/20493

**Published:** 2020-07-14

**Authors:** Aravind Sesagiri Raamkumar, Soon Guan Tan, Hwee Lin Wee

**Affiliations:** 1 Saw Swee Hock School of Public Health National University of Singapore Singapore Singapore; 2 Department of Pharmacy National University of Singapore Singapore Singapore

**Keywords:** health belief model, physical distancing, COVID-19, text classification, deep learning, recurrent neural network, social media

## Abstract

**Background:**

Public health authorities have been recommending interventions such as physical distancing and face masks, to curtail the transmission of coronavirus disease (COVID-19) within the community. Public perceptions toward such interventions should be identified to enable public health authorities to effectively address valid concerns. The Health Belief Model (HBM) has been used to characterize user-generated content from social media during previous outbreaks, with the aim of understanding the health behaviors of the public.

**Objective:**

This study is aimed at developing and evaluating deep learning–based text classification models for classifying social media content posted during the COVID-19 outbreak, using the four key constructs of the HBM. We will specifically focus on content related to the physical distancing interventions put forth by public health authorities. We intend to test the model with a real-world case study.

**Methods:**

The data set for this study was prepared by analyzing Facebook comments that were posted by the public in response to the COVID-19–related posts of three public health authorities: the Ministry of Health of Singapore (MOH), the Centers for Disease Control and Prevention, and Public Health England. The comments made in the context of physical distancing were manually classified with a Yes/No flag for each of the four HBM constructs: perceived severity, perceived susceptibility, perceived barriers, and perceived benefits. Using a curated data set of 16,752 comments, gated recurrent unit–based recurrent neural network models were trained and validated for text classification. Accuracy and binary cross-entropy loss were used to evaluate the model. Specificity, sensitivity, and balanced accuracy were used to evaluate the classification results in the MOH case study.

**Results:**

The HBM text classification models achieved mean accuracy rates of 0.92, 0.95, 0.91, and 0.94 for the constructs of perceived susceptibility, perceived severity, perceived benefits, and perceived barriers, respectively. In the case study with MOH Facebook comments, specificity was above 96% for all HBM constructs. Sensitivity was 94.3% and 90.9% for perceived severity and perceived benefits, respectively. In addition, sensitivity was 79.6% and 81.5% for perceived susceptibility and perceived barriers, respectively. The classification models were able to accurately predict trends in the prevalence of the constructs for the time period examined in the case study.

**Conclusions:**

The deep learning–based text classifiers developed in this study help to determine public perceptions toward physical distancing, using the four key constructs of HBM. Health officials can make use of the classification model to characterize the health behaviors of the public through the lens of social media. In future studies, we intend to extend the model to study public perceptions of other important interventions by public health authorities.

## Introduction

### Background

The Health Belief Model (HBM) is a theoretical model constructed based on psychological and social theory [1]. It has been widely used as a conceptual framework in behavioral research to understand the health behavior of individuals. The HBM attempts to explain and predict behavioral outcomes based on two main aspects: the desire to avoid a health threat (ie, infection or illness) and the perception of the effectiveness of the behavior adopted to counteract that threat. The perception of threat is composed of an individual’s perceived susceptibility and perceived severity to a specific illness or threat. The effectiveness of a specific health behavior is dependent on the interaction between the perceived benefit of the behavior and the perceived barriers to taking action to mitigate the threat or illness [2]. In addition, cues to action are prompts or events that trigger the health behavior of interest. Cues to action can be divided into internal (eg, physical symptoms) or external (eg, mass media, reminders, advice) triggers. Lastly, health motivation (or self-efficacy) explains how predisposed an individual is to respond to cues to action based on the value of their health. The HBM has been adopted as an explanatory model of the communication process [3]. Constructs of the HBM have been used to study the health beliefs of the public on the social media platform Twitter [4] and analyze responses to outbreak communication campaigns on Instagram [5].

In the context of the ongoing coronavirus disease (COVID-19) outbreak, the constructs of the HBM will be influenced by the interaction of information from news and media reports, government policy actions, and feedback from the public throughout the course of the outbreak. These messages will alter an individual’s behavior if it targets perceived barriers, benefits, self-efficacy, and threat. One such example is the physical distancing measures put forth by public health authorities across the globe. Physical distancing measures constitute a combination of measures that aim to increase the physical distance between individuals and reduce the frequency of close contact, which results in lower community transmission of the virus. We note the distinction between physical distancing and self-isolation measures and quarantine orders. Isolation and quarantine measures are for individuals who display COVID-19–related respiratory symptoms or have had close contact with confirmed or suspected cases [6]. For the physical distancing measure, public behavior can either be supportive (desired) or critical (undesired).

The perceptions of the public toward physical distancing can be ascertained by mining the relevant content from social media platforms. Public health authorities have been using Facebook and Twitter to post regular updates about COVID-19 through their official pages or accounts [7]. Members of the public respond to these updates through comments or tweets. Their opinions may be neutral, supportive, or critical. It is practically difficult for public health authority officials to manually analyze the content on social media on a periodic basis. Automated analysis of textual content can be facilitated through machine learning methods such as text classification or categorization. Such methods can be used to dynamically classify bulk social media content for real-time analysis so that public health authority officials can gauge the public response to their health messages. In a related study, a deep learning–based text classification model was used to classify tweets about the human papillomavirus vaccine with the HBM constructs [4]. Through the study, it was possible to identify the time periods during which the different HBM constructs were prevalent.

### Study Overview

In this study, using the constructs of the HBM, we aimed to develop deep learning–based text classification models for classifying social media content posted in response to the COVID-19 updates of public health authorities. The models were tailored specifically for content related to the physical distancing intervention. We used the gated recurrent unit (GRU) variant of the recurrent neural network (RNN) [8] to build the text classification models. The models were trained and validated with a data set of 16,752 comments primarily extracted from the Facebook pages maintained by public health authorities in Singapore, the United States, and England. As a demonstrative case study for testing, we used the model to classify all Facebook comments received in response to the COVID-19 Facebook posts of the Ministry of Health, Singapore (MOH) during the first quarter of 2020. In addition, we created an online demo webpage for bulk classification of social media data (Facebook comments, tweets) related to physical distancing using the models developed in this study.

## Methods

### Data Set Preparation

Data for this study were extracted from three Facebook pages using the Facepager tool [9] for the time period from January 1 to March 31, 2020. The three Facebook pages are officially managed by MOH Singapore [10], the Centers for Disease Control and Prevention (CDC) in the United States [11], and Public Health England (PHE) [12]. Extracted data included posts by public health authorities and comments on those posts. From the extracted posts, COVID-19 posts were identified by searching the posts for the existence of at least one of the keywords “wuhan virus,” “coronavirus,” “ncov,” “ncov-2019,” “covid,” and “covid-19.” The comments received on the filtered COVID-19 posts were subsequently classified using four key HBM constructs: perceived susceptibility, severity, benefits, and barriers. We focused on the physical distancing intervention as the preventive behavior of interest. In Table 1, definitions and sample comments for the HBM constructs are provided in the context of this study.

**Table 1:** Definition of the Health Belief Model constructs examined and sample comments in relation to coronavirus disease.

Construct	Definition
Perceived susceptibility	Comments that indicated an assessment of the increased likelihood of contracting coronavirus disease, highlighting increasing local prevalence and the high number of imported cases
Perceived severity	Comments that indicated an assessment of an increase in the perceived seriousness and consequences of contracting coronavirus disease (eg, hospitalization, pneumonia, death, mortality risk)
Perceived benefits	Comments that supported physical distancing measures (eg, school closure, working from home, cancellation of events and mass gatherings) to reduce the transmission of coronavirus disease
Perceived barriers	Comments that mentioned the difficulties, challenges, and negative effects of physical distancing (eg, loss of freedom, violation of individual rights, inconvenience, loss of income), as well as the perceived ineffectiveness of physical distancing

The classification of comments for perceived susceptibility and perceived severity was performed using a rule-based filtering method where we used a set of candidate keywords that accurately represented these two constructs. Comments that met the filtering criteria were flagged accordingly. However, this approach did not work well for perceived barriers and perceived benefits as we could not find an accurate set of keywords that represented these constructs. Hence, the comments were manually classified with the help of two coders. All comments were manually validated using the abovementioned approaches. Interrater agreement between the two coders was strong. Cohen scores were 0.91, 0.86, 0.89, and 0.91 for the four HBM constructs of perceived susceptibility, perceived severity, perceived benefits, and perceived barriers, respectively. After eliminating blank comments and comments with images, we arrived at a total of 99,197 comments. However, only 8376 comments (8.44%) represented at least one of the four HBM constructs.

The next step was to prepare a balanced data set from the analyzed comments to train and validate the text classification models. All 8376 comments that represented at least one of the four HBM constructs were first added to the data set. Next, another 8376 comments which did not represent any of the four HBM constructs were added. As a result, the final data set was comprised of 16,752 comments with 50% of the comments representing preventive behavior (any of the HBM constructs). The comments from this data set were randomly divided into training (n=13,401) and validation (n=3351) sets using the traditional 80/20 split method. Sample comments representing the HBM constructs are provided in Multimedia Appendix 1.

### Text Classification Model

**Figure 1:**
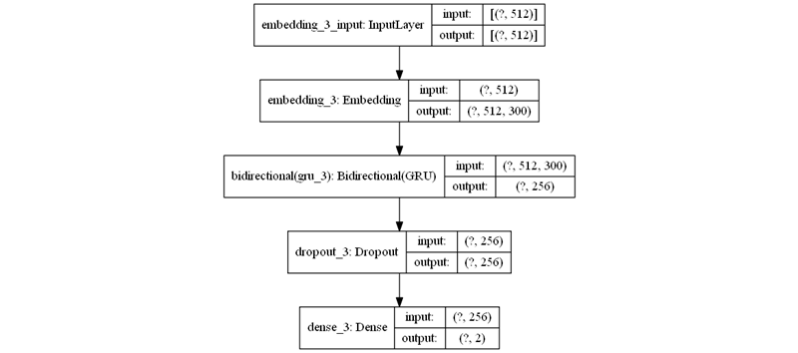
Health Belief Model text classifier neural network architecture. GRU: gated recurrent unit.

For the first time, we used a GRU-based RNN model [8] to classify content using the HBM constructs. The GRU model is considered an improvement over the basic RNN model [13] as it addresses the vanishing gradient problem. The gradients carry information used in the updates to the RNN parameter; when the gradients become progressively smaller, the parameter updates become insignificant. As a result, no real learning is performed. Hence, the learning of long data sequences is hampered due to vanishing gradients. Conversely, GRU makes use of the update gate and reset gate to solve this issue [8]. RNN was previously used in HBM-based models to study tweets [4]. A bidirectional structure was set for the GRU model as it helps record information from both backward and forward states in the neural network [14]. An embedding layer was used as the first layer of the model. The embedding layer is useful for mapping words to a vector of continuous numbers. For this purpose, we used pretrained GloVe (Global Vectors for Word Representation) word vectors [15], which map each word to a vector of a specific size. The classification models were implemented in TensorFlow 2.0 (Google Brain, Google Inc) [16] and comprised five layers, as well as a dropout layer added to avoid overfitting [17]. Accuracy and binary cross-entropy loss were the metrics used to evaluate the performance of the models. Other parameters set for the models were as follows. Sequence length, embedding size, vocabulary size, and number of units were set to 512, 300, 50,000, and 128, respectively. Adam optimizer was used as the optimization algorithm in the models [18]. In Figure 1, the common architecture of the classification models is illustrated. For each of the four HBM constructs, the model was separately trained and validated. As a result, we obtained four binary classification models with a common design.

## Results

### Classification Performance

In Table 2, the training and validation performance of the models are depicted in the form of mean accuracy and mean loss calculated from six epochs, along with the standard deviation values. All four models had an accuracy above 0.91 for both the training and validation sets. In the training set, perceived severity had the best accuracy (µ=0.95), followed by perceived barrier (µ=0.94), perceived susceptibility (µ=0.93), and perceived benefit (µ=0.91). The validation accuracy values were similar; perceived susceptibility (µ=0.92) was the exception. Through the epochs, the losses gradually reduced for the constructs in both the training and validation cycles. Multimedia Appendix 2 illustrates the loss values by epoch for training and validation.

**Table 2:** Health Belief Model classification models’ performance statistics.

Health Belief Model construct	Training accuracy, mean (SD)	Training loss, mean (SD)	Validation accuracy, mean (SD)	Validation loss, mean (SD)
Perceived susceptibility	0.93 (0.04)	0.17 (0.09)	0.92 (0.03)	0.23 (0.15)
Perceived severity	0.95 (0.02)	0.14 (0.07)	0.95 (0.02)	0.11 (0.03)
Perceived benefit	0.91 (0.03)	0.20 (0.07)	0.91 (0.01)	0.22 (0.01)
Perceived barrier	0.94 (0.01)	0.15 (0.04)	0.94 (0.00)	0.15 (0.01)

### MOH Case Study

The HBM classification models were used to classify all comments received on COVID-19 posts by the MOH in the first quarter of 2020. We chose the MOH as a case study because it was the most active in posting on Facebook among the three public health authorities discussed in this study. In total, 9053 comments were classified as part of this exercise. In Table 3, the specificity, sensitivity, and balanced accuracy percentages are listed for the four HBM constructs. Specificities were above 96% for all four constructs, and perceived susceptibility and perceived barrier had the highest values (99.7% and 99.0%, respectively). However, these two constructs had the lowest sensitivities (79.6% and 81.5%) among the four constructs, indicating that the corresponding models overpredicted false-negative cases. Due to skewed specificities, the models for classifying perceived susceptibility and perceived barrier achieved a balanced accuracy of 89.6% and 90.3%, respectively. On the other hand, both sensitivity and specificity were above 90.0% for perceived severity and perceived benefit. Hence, the balanced accuracy for these two constructs was high, with values of 96.5% and 93.7%.

The performance of the classification models was calculated with the following equations: SP=TP/(TP+FN); SE=TN/(TN+FP); and BA=(SP+SE)/2, where SP is specificity, TP is true positives, FN is false negatives, SE is sensitivity, TN is true negatives, FP is false positives, and BA is balanced accuracy.

**Table 3:** Performance of the Health Belief Model classification models with MOH Facebook comments.

Health Belief Model construct	Specificity, %	Sensitivity, %	Balanced accuracy, %
Perceived susceptibility	99.7	79.6	89.6
Perceived severity	98.8	94.3	96.5
Perceived benefit	96.5	90.9	93.7
Perceived barrier	99.0	81.5	90.3

**Figure 2:**
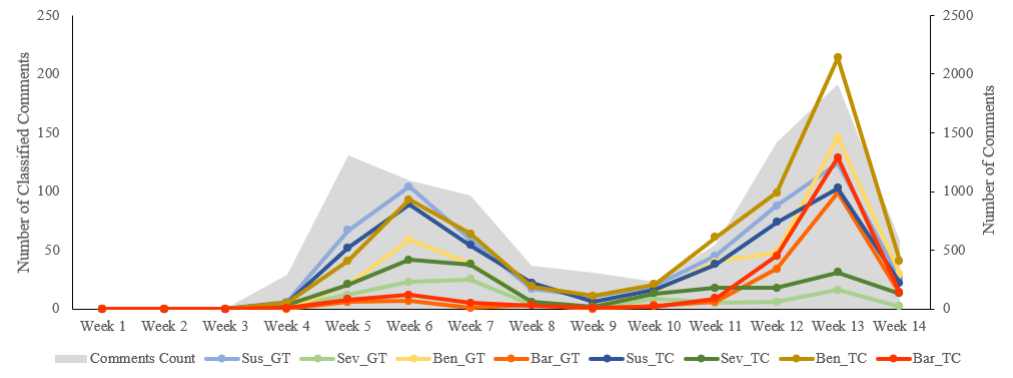
Classification of Ministry of Health comments with Health Belief Model constructs. The primary x-axis is for the classified comments count for the Health Belief Model constructs, while the secondary x-axis is for the total comments count. Sus refers to perceived susceptibility, Sev refers to perceived severity, Ben refers to perceived benefit, and Bar refers to perceived barrier. Suffixes GT and TC refer to ground truth and text classification, respectively.

In Figure 2, the number of classified comments per HBM construct is plotted as a line graph to compare the ground truth (manually classified comments) with the deep learning classification results for the HBM constructs. The total number of comments is plotted as an area graph to facilitate the interpretation of the prevalence of the HBM constructs. The data has been aggregated at the week level to facilitate interpretation. Until the end of Week 4 (January 25, 2020), the number of comments representing the four HBM constructs was low. This is primarily because the total comments were also low. There were two peaks periods in the prevalence of the HBM constructs, one in Week 6 (February 2-8) and the other in Week 13 (March 22-28). Except for perceived susceptibility, the classification models seem to overpredict compared to the ground truth. The gap between the actual results and predicted results is evident for perceived benefits in both peak periods. Overall, the proportion of comments on perceived severity and perceived barriers was low, with only 12.4% and 6.3% prevalence, respectively. Conversely, perceived benefits and perceived susceptibility accounted for 20.5% and 17.5% of the total comments, respectively.

## Discussion

The similarity in training and validation mean accuracy rates indicates that overfitting and underfitting aspects were minimal, thereby supporting our strategy of creating a data set with equal percentages of preventive behavior comments and nonpreventive behavior comments. The variable length of comments could be an issue, as we noticed that models performed well with longer comments as the context is more discernable. In the case study with MOH Facebook comments, the developed classification models achieved better specificities, sensitivities and accuracies than were achieved in a previous study [4] where a deep learning model was used to classify tweets with HBM constructs. However, the slightly lower sensitivities of perceived susceptibility and perceived barriers resulted in more false-negative cases during classification. The high specificities for all four models were a result of the skewed nature of the data, since only 8.4% of comments in the base set represented at least one of the HBM constructs. Sensitivity is more important than specificity in this study since positive cases need to be more accurately predicted.

The comparison of ground truth with the classification results at the week level indicates that the classification models predict upward and downtrend trends in a precise manner. There are two peak periods in the prevalence of HBM constructs among comments. The first peak period corresponded to the week when Singapore shifted to Disease Outbreak Response System Condition (DORSCON) orange, the second-highest level of alert for disease outbreaks in Singapore, on February 7, 2020 [19]. However, the prevalence of perceived barrier comments did not resemble the other three HBM constructs in this first peak period. The second peak in the prevalence of HBM constructs did not correspond to any discernible real-world event; we speculate that MOH Facebook page followers started commenting at a higher frequency from this week. Since our data collection period ended on March 31, 2020, Week 14 does not include a full week of data. In this second peak, the prevalence of perceived barriers increases considerably to indicate that the public started talking about barriers to physical distancing at a discernible level during this period. At the same time, the prevalence of perceived severity remained consistently low and did not increase during the second peak.

In the first 13 weeks of 2020, it can be deduced that people talked more about susceptibility and the benefits of physical distancing than severity and barriers. Overall, the prediction results closely followed the ground truth with no outlying trends, thereby indicating that the classification models can be used to predict trends in the HBM constructs in the upcoming months in the context of physical distancing interventions. We have created an online demonstration webpage to showcase the bulk classification of social media content using the developed models [20]. We converted the text classification models to the TensforFlow.js format for this purpose [21]. To enable programmatic usage and retraining with new data, the original and converted files of the four classification models have been made available in the HDF5 (Hierarchical Data Format version 5) and TensorFlow.js formats, respectively [22].

This study has certain limitations. The comments analyzed in this study should be considered a snapshot of the overall public response, as users can delete comments from Facebook retrospectively. The opinions of Facebook users regarding physical distancing could be different on Facebook pages other than the public health authority page of their respective country. Those opinions are not covered in this study. The rule-based filtering approach for the manual classification of comments may not be able to accurately capture all the comments under each of the respective HBM constructs. Spelling mistakes, memes, colloquial words, and non-English comments expressing a certain health belief may not be captured.

In conclusion, this study showed that our deep learning–based text classifiers successfully yielded accurate classifications of COVID-19 Facebook comments using the HBM constructs, in the context of the physical distancing intervention. This further demonstrates the potential for developing deep learning prediction systems to classify big data from social media using behavioral models and frameworks. We hope that the classification model files from this study and the bulk classifier demonstration webpage are of practical use for public health officials and the scientific community. In future work, we intend to further improve the classification models and extend our study through various approaches. First, variable-length comments should be handled more efficiently. Second, we intend to experiment with a two-stage classification approach, where the first-stage classification predicts whether a comment represents a preventive behavior or not. The second-stage classification would then predict whether a filtered comment represents any of the four HBM constructs. Third, we intend to study social media users’ perceptions toward other public health authority interventions, such as wearing face masks.

## Appendix

Multimedia Appendix 1 Sample comments representing the Health Belief Model constructs.

Multimedia Appendix 2 Loss values of the training and validation models for the four HBM constructs. Sus refers to perceived susceptibility, Sev refers to perceived severity, Ben refers to perceived benefit, and Bar refers to perceived barrier. Suffixes T and V refer to training and validation sets, respectively. HBM: Health Belief Model.

## Abbreviations

CDC: Centers for Disease Control and Prevention (United States of America)
COVID-19: coronavirus disease
DORSCON: Disease Outbreak Response System Condition
GloVe: Global Vectors for Word Representation
GRU: gated recurrent unit
HBM: Health Belief Model
MOH: Ministry of Health (Singapore)
PHE: Public Health England
RNN: recurrent neural network
