# Supplementary material for: Use of Health Belief Model–Based Deep Learning Classifiers for COVID-19 Social Media Content to Examine Public Perceptions of Physical Distancing: Model Development and Case Study
Source: JMIR Public Health Surveill. 2020 Jul 14;6(3):e20493. doi: 10.2196/20493 (PMC7363169; doi:10.2196/20493)
Supplement: Multimedia Appendix 1 [file publichealth_v6i3e20493_app1.docx]

## Multimedia Appendix 1

**Table A1.** Sample classified comments representing perceived susceptibility.

| Sl. No | Facebook Comment |
| --- | --- |
| 1 | From what I see this virus is a type of pneumonia, more deadly, and contagious than that. Shouldn't the vaccine that are out there reduce the severity, or treatment be something close to it. I am at high risk of it and terrified to even go out shopping for essential things. I want a chance of surviving this. |
| 2 | Why aren't they talking about asymptomatic carriers?! It's not just people with symptoms that are spreading it and they KNOW that. ?????? |
| 3 | As feedback, some parents have, by choice, have decided to take their children off school in fear of how far socially irresponsible cases have spread this. Furthermore, while we can understand why closing schools will affect working parents, there was advise for companies to try telecommuting, and working from home to control crowding and promote social distancing. Doesn't this in some way, allow a working parent the ability to be home? |
| 4 | Why are we not implementing widespread testing? Waiting for symptons to appear and for people to step forward is not enough given the increase in community spread. |
| 5 | no not a hospital, a tax office. It’s ridiculous 😡 but he has been told he’s to go. And if that site gets closed he will be moved to another. Public health is more important 😡 |

**Table A2.** Sample classified comments representing perceived severity.

| Sl. No | Facebook Comment |
| --- | --- |
| 1 | Now they are saying it could be 100k deaths or more! So scary! |
| 2 | I am more worried about getting the disease and dying and leaving my babies by their selves |
| 3 | Health experts have said social distancing and increased awareness resulting from the coronavirus pandemic helped end Hong Kong’s winter influenza season nine weeks earlier this time than last year, but 113 people still died of the flu. The number of deaths in the city more than halved from 356 in 2019, while intensive care unit admissions dropped from 601 to 182. |
| 4 | cnfirmed sign of community spread , school is the biggest cluster and most vulnerable one , what's the gov is waiting for before closing schools? when the case curve spikes to a certain level? pls be reminded this is life and death , not cold number or graphic . pls do not be over confident with the contact tracing method, it's proved not working any more |
| 5 | Because we are only recording cases ALREADY serious enough to be admitted to hospital, therefore more likely to die. If you don't bother testing or counting people with only mild symptoms, then of course your death rate looks worse. |

**Table A3.** Sample classified comments representing perceived benefits.

| Sl. No | Facebook Comment |
| --- | --- |
| 1 | Psychologist Baruch Fischhoff, who studies decision-making, among other things, says it's fine to go outside, to go anywhere outdoors really, so long as you're committed to following the social distancing protocols as outlined by medical experts. |
| 2 | Hygiene in washing hands with soap, prevent spread wear masks to manage the prevention. Ensure the masks are N95 or 3-4 ply surgical masks. Keep a distance of 2 mtrs from people as a form of cooperation in physical distancing ( social distancing). |
| 3 | “Stricter Safe Distancing “ how about the public transportation, I wondered if ours working hours can be rescheduled to 3 batches base on locations of examples 08:00-16:00 ,09:00-17:00, 10:00-18:00 and will this be help to reduce the crowd of the public transportation and the eating places |
| 4 | But meanwhile I do understand we need to keep the society running and can’t take extreme measures. Just hope that some unnecessary gatherings can be banned. |
| 5 | Stay at home to save a seniors life 👍 |

**Table A4.** Sample classified comments representing perceived barriers.

| Sl. No | Facebook Comment |
| --- | --- |
| 1 | If you care about your family stay home or isolate yourself in the home while you go out work. Not everyone can stay home as will starve to death and go bankrupt! This is real life.... |
| 2 | Due to the situation with the coronavirus, people who are more at risk like older adult, people with allergies, diabetics, etc., should be commanded and protected by law, so they don't lose their job, to stay home until all of this pass. |
| 3 | Non of this matters if people are still able to flock to stores like home depot and lowes. There is no regulation on how many people go in the store. The parking lots are full like black friday. Someone needs to regulate these stores as they stay open for essential items. |
| 4 | 1m Apart in queue is not working, at least not working at food courts, kopitiam I visited today. Even when ppl are in queue standing 1m apart on the marked floor, Sellers/Cashiers kept urging people to move forward to take their orders and payment in order to clear the queue fast. WHAT'S THE POINT THEN? |
| 5 | Stay at home ,but people who are self employed. What they can do ? |
