# Supplementary figures and images for: Use of Health Belief Model–Based Deep Learning Classifiers for COVID-19 Social Media Content to Examine Public Perceptions of Physical Distancing: Model Development and Case Study
Source: JMIR Public Health Surveill. 2020 Jul 14;6(3):e20493. doi: 10.2196/20493 (PMC7363169; doi:10.2196/20493)

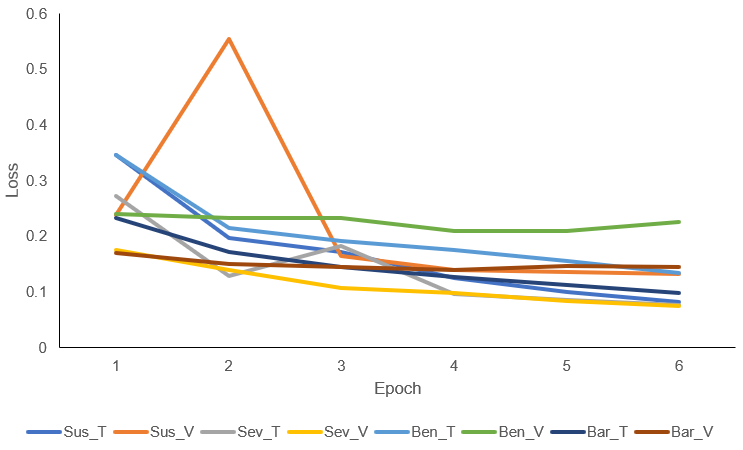

Supplement: Multimedia Appendix 2 [file publichealth_v6i3e20493_app2.PNG]
